# Fungi Associated with the Hemlock Woolly Adelgid, *Adelges tsugae*, and Assessment of Entomopathogenic Isolates for Management

**DOI:** 10.1673/031.010.6201

**Published:** 2010-06-11

**Authors:** W.R. Reid, B.L. Parker, S.Y. Gouli, M. Skinner, V.V. Gouli, H.B. Teillon

**Affiliations:** Entomology Research Laboratory, Department of Plant and Soil Sciences, University of Vermont, 661 Spear St., Burlington, VT 05405-0105.

**Keywords:** bioassay, biological control, entomogenous, mycoinsecticides, *Tsuga canadensis*, *Tsuga caroliniana*

## Abstract

Fungi associated with the hemlock wooly adelgid, *Adelges tsugae* Annand (Hemiptera: Adelgidae), were collected throughout the eastern USA and southern China. Twenty fungal genera were identified, as were 79 entomopathogenic isolates, including: *Lecanicillium lecanii* (Zimmermann) (Hypocreales: Insertae sedis), *Isaria farinosa* (Holm: Fries.) (Cordycipitaceae), *Beauveria bassiana* (Balasamo) (Hyphomycetes), and *Fusarium* spp (Nectriaceae). The remaining fungal genera associated with insect cadavers were similar for both the USA and China collections, although the abundance of *Acremonium* (Hypocreaceae) was greater in China. The entomopathogenic isolates were assayed for efficacy against *Myzus persicae* (Sulzer) (Homoptera: Aphididae) and yielded mortality ranging from 3 to 92%. Ten isolates demonstrating the highest efficacy were further assessed for efficacy against field-collected *A. tsugae* under laboratory conditions. Overall, two *B. bassiana*, one *L. lecanii*, and a strain of *Metarhizium anisopliae* (Metchnikoff) (Hypocreales: Clavicipitaceae), demonstrated significantly higher efficacy against *A. tsugae* than the others. Isolates were further evaluated for conidial production, germination rate and colony growth at four temperatures representative of field conditions. All isolates were determined to be mesophiles with optimal temperature between 25–30° C. In general, conidial production increased with temperature, though two *I. farinosa* produced significantly more conidia at cooler temperatures. When efficacy values were compared with conidial production and temperature tolerances, Agricultural Research Service Collection of Entomopathogenic Fungi (ARSEF) 1080, 5170, and 5798 had characteristics comparable to the industrial *B. bassiana* strain GHA.

## Introduction

The hemlock woolly adelgid, *Adelges tsugae* Annand (Hemiptera: Adelgidae), is native to China and is a pest of eastern hemlock, *Tsuga canadensis* (*L*.) Carriere (Pinales: Pinaceae), and Carolina hemlock, *Tsuga caroliniana* Englem., in the eastern USA (Knauer et al. 2002) that causes tree mortality (Orwig and Foster 1998). *A. tsugae* has a hemimetabolous life cycle, spending most of its life on hemlock. When feeding, it inserts its mouthparts directly into the tree, and the insect remains in this position throughout its life. Many studies regarding the biology, physiology, and ecology of this insect have been published, (McClure 1987, 1990, 1991; Young et al. 1995; Parker et al. 1997, 1999; Gouli et al. 2000; Skinner et al. 2003) and various management tactics have been investigated (McClure 1987, 1992; Cheah and McClure 1996; Montgomery 1996; Sasaji and McClure 1997; Wallace and Hain 2000; Blumenthal 2002; Cassagrande et al. 2002). These tactics, however, are limited by the cost of treating large areas and by the prevalence of hemlock in watershed regions where use of broad spectrum chemical insecticides is generally forbidden. Currently, no effective management strategy has been found that is amenable to large-scale application in these watershed regions. The outcome of releases of the predacious lady beetle, *Sasajiscymnus tsugae* require 4–7 years for assessment, and results vary based on the initial quality of the test site (Cheah 2004). As tree mortality can occur in as little as three years (McClure 1987) and performance of *S. tsugae* is superior when released in healthier hemlock forests (Cheah 2004), management tactics that provide immediate protection of hemlocks are needed. These tactics must be relatively inexpensive and easy to adopt on a large scale. Insect pathogens represent an environmentally sound approach to pest management that meets these requirements, but due to the feeding behavior of *A. tsugae*, only those that cause mortality via direct contact, such as fungi, are suitable. Naturally-occurring fungal pathogens of *A. tsugae* have been identified and recovered, several of which were found to induce 64–82% mortality among the adult sistens when applied at a rate of 1 × 10^8^ conidia per ml (Gouli et al. 1997). Moreover, research has shown that several fungal isolates are pathogenic to *A. tsugae*, but do not cause significant mortality to *S. tsugae* (Parker et al. 2004). The fungi previously obtained by Gouli et al. (1997) were acquired from a single location in the eastern USA. The objectives of this study were to expand on that work by isolating additional fungal entomopathogens from *A. tsugae* in the eastern USA and China and to characterize them for suitability as mycoinsecticides.

**Table I.**
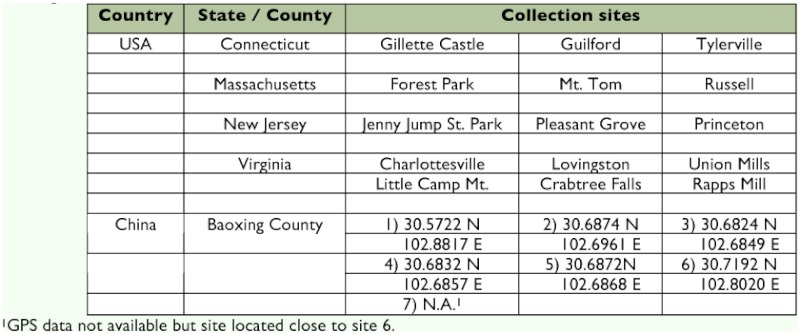
Locations of *Adelges tsugae* populations sampled from hemlock in the eastern United States and locations in Baoxing, China.

## Materials and Methods

### Sampling of *A. tsugae*

Collections of *A. tsugae* were conducted in the eastern USA during the spring and fall of 1997 (Table 1). Sample sites consisted of multi-aged hemlock trees with new growth and moderate infestations of *A. tsugae.* Within each site, 10 infested trees ranging in height from 3–13 m were selected, and, from each tree, ten 10-cm branchlets were collected and singly placed in plastic bags for a total of 1000 linear cm of infested hemlock branchlets per site. Samples were held in the laboratory at 15–25° C and processed within 72 h of collection. In China, *A. tsugae* is not a serious pest of hemlock and was difficult to find. Therefore, forest stands with hemlock trees were exhaustively searched in a radial pattern, sampling every tree with signs of *A. tsugae.* All available samples on a given tree were collected, up to 25 branchlets per tree.

### Specimen processing, fungal identification and storage

Branchlets containing *A. tsugae* were examined at 40× magnification, and individuals with signs of fungal infection (eg. off-color, misshapen, bloated, or mummified) were removed from the twig with fine-point forceps and transferred to sterile paper towels moistened with sterile distilled water containing 30 IU of penicillin G and 70 IU of streptomycin sulfate. Forceps were disinfected between cadavers with 75% ethanol to prevent cross contamination. Cadavers were held at 22 ± 2° C for 1–2 wks until fungal outgrowth was observed. Fungal outgrowth was collected using a sterile probe and transferred to potato dextrose agar medium containing 150 IU/ml penicillin G and 350 IU/ml streptomycin sulfate. Cultures were incubated at 22 ± 2° C for 7 d, identified at 400× magnification using the methodology of Gouli et al. (2005), and transferred to -80° C storage in the Entomology Research Laboratory fungal collection at the University of Vermont, Burlington, VT. All isolates were also submitted to the USDA Agricultural Research Service collection of Entomopathogenic Fungi (ARSEF), Ithaca, NY for verification of identification and for preservation.

### Test fungi and preparation for bioassays

Fungi used in this study were obtained as multispore isolates from the *A. tsugae* cadavers collected in the eastern USA and China. In addition, isolates previously obtained from *A. tsugae* in the eastern USA were included (Gouli et al. 1997). As reference standards, the study included two additional isolates: GHA, a *Beauveria bassiana* (Balasamo) (Hyphomycetes) strain (BotaniGard) from Laverlam International Corporation (www.laverlamintl.com), and ARSEF 1080, a *Metarhizium anisopliae* (Metchnikoff) (Hypocreales: Clavicipitaceae), originally isolated from *Helicoverpa zea* (Boddie) (Lepidoptera: Noctuidae) in Florida. For the remainder of this article, all isolates, with the exception of GHA, are referred to by their ARSEF accession number. Stock plates of fungi were prepared as spread plates on ¼ strength Sabouraud's dextrose agar supplemented with 1% yeast extract (SDAY/4) and maintained at 4° C until needed. Fungal material used for testing was obtained by subculturing from the stock plates onto SDAY/4. Cultures were prepared as spread plates and incubated for 10 d at 22° C. Conidial suspensions were prepared by transferring the fungal colonies from two Petri dishes into 20 ml of sterile distilled water containing 0.5 g of glass balls followed by vigorous shaking.

Suspensions were filtered through eight layers of cheesecloth and calibrated to a stock concentration of 1 × 10^8^ conidia per ml using a Neubauer haemocytometer. Additional test concentrations were prepared through serial dilutions to 1 × 10^7^, 5 × 10^6^, 1 × 10^6^, 1 × 10^5^ and 1 × 10^4^ conidia per ml. For all assays, the viability of the testing material was assessed post-application by spraying a 9 cm diameter Petri dish containing 20 ml SDAY/4 for 2–3 s with the suspension containing 5 × 10^6^ conidia per ml. These plates were incubated at 25° C for 20 h (16 h for GHA), after which three drops of lactophenol cotton blue stain (VWR International, www.vwr.com) were applied to the medium surface to kill the fungi. Glass coverslips were placed over the medium, and conidia were inspected for germination at each of the three spots at 400× magnification. A conidium was considered germinated if the germ tube was longer than the width of the conidium (Hywell-Jones and Gillespie 1990). All fungal material used in this study had germination rates > 95%.

### *Myzus persicae* bioassays

A stock laboratory colony of apterous *Myzus persicae* (Sulzer) (Homoptera: Aphididae) was maintained on mustard, *Brassica juncea* (L.) Czernajev and Cosson (Brassicales: Brassicaceae), cv. Florida broad leaf. Five adult females were placed on a freshly excised fourth or fifth true leaf for 24 h, during which time they produced first instars. The number of first instars was adjusted to 12 per leaf, and, throughout the experiment, the leaf petioles were held in a covered, 30 ml plastic creamer cup filled with a 60 ppm solution of 20-10-20 allpurpose fertilizer (Peter's Professional Fertilizer, Scotts-Sierra Horticultural Products). Cups were then incubated in a plastic tray fitted with a thrips-proof mesh lid, to allow for air exchange. Aphids were incubated at 22 ± 2° C for 7 d, at which point all insects were 1-d-old adult females. Five leaves for each fungal replicate were sprayed on both sides with 0.6 ml of a 5 × 10^6^ conidia per ml suspension using an airbrush at 12 psi (Badger Airbrush Co., www.badgerairbrush.com). Leaves were individually sprayed to the point of run-off. These assays were replicated a single time and the results were used to roughly screen which isolates were pathogenic. Equal numbers of controls were treated with sterile distilled water. All treatments were held in an incubator at 22 ± 2° C and 16:8 L:D for 6 d. Mortality was assessed daily, and new birthed juveniles were removed to maintain a constant number of aphids on each leaf.

### *Adelges tsugae* bioassays

Populations of *A. tsugae* were fieldcollected from *T. canadensis* in Lovingston, VA. Infested branchlets were collected from 10 codominant trees, 6–8 m tall, that had never been treated with insecticides. These trees had new growth, which supports populations of *A. tsugae* with high vigor (McClure 1991). Branchlets with ≥ 2.5 cm of new growth and at least 24 first instar aestivating adelgids were selected as experimental units. This stage of *A. tsugae* was chosen for testing because the insect remains in this form for several months without molting and not covered with the waxy exudate, making it an ideal target for fungi. Five branchlets were treated for each replicate at each concentration. Branchlets were individually sprayed on both sides with 0.6 ml of test conidial suspension using an airbrush at 12 psi. Spraying was conducted so that an even mist of suspension was applied, but so that the suspension did not run off the branchlet. Controls were likewise sprayed with sterile distilled water. Treated branchlets were held singly in Pyrex test tubes (20 × 250 mm) containing 20 g of white sand that had been previously heated for 5 h at 85° C. Four ml of sterile distilled water were added to the sand in each tube to maintain branchlet viability (Gouli et al. 1997; Parker et al. 1999). The tops of the tubes were covered with one layer of muslin (140 thread count) held in place with an elastic band. Tubes were held at 22 ± 2° C, and mortality was assessed 6 d post-application. *A. tsugae* were considered dead if they did not maintain their body turgor after gentle probing with a blunt needle or if they were solid with fungal mycelium (Parker et al. 1999). Experiments were conducted as completely randomized designs and replicated three times. The first 12 dead *A. tsugae* observed from each treatment were transferred to Petri dishes lined with sterile paper towels moistened with sterile distilled water containing 30 IU of penicillin G and 70 IU of streptomycin sulfate. Dishes were incubated at 22 ± 2° C and examined for fungal outgrowth after 5 d.

### Characterization of fungi for growth and sporulation

The 12 isolates tested in the *A. tsugae* bioassays were assessed for rate of growth, conidial production and germination at 15, 20, 25, and 30° C. For each, 10 µl of a suspension of 1 × 10^6^ conidia per ml was inoculated onto a six mm diameter sterile filter paper placed in the center of a standard nine cm diameter Petri dish containing 20 ml of SDAY/4. Dishes were inverted and incubated in the dark for 20 d. Two orthogonal measurements of the colony diameter were recorded on days 5, 10, 15, and 20, and averaged for each time point. At 20 d, four sample cores were taken from these colonies using a 5 mm diameter cork borer. These were pooled into 10 ml of 0.1% Tween 80 containing 0.6% Greenshield (Whitmire Micro-Gen Research Labs, www.wmmg.com/home.asp) and sonicated for 10 min to separate conidia. Conidia concentrations were estimated using two counts on a Neubauer improved haemocytometer at 400× magnification and adjusted to conidia per cm2 of colony. For the assessment of conidial germination rate, 50 µl of a suspension of 1 × 10^6^ conidia per ml for each isolate was streaked onto 9 cm diameter Petri dishes containing one-tenth strength Sabouraud's dextrose agar supplemented with 0.10% yeast extract (SDAY/10). All germination was conducted in the dark and was assessed at 10, 13, 15, and 17 h after streaking. Germination was assessed as previously described.

### Experimental designs and statistics

For all bioassays, data were corrected for control mortality using Abbott's correction factor (Abbott 1925). A Kolmogorov-Smirnov test for non-normality was applied using PROC FREQ in SAS (SAS Institute 1996) to test the distribution of mortality within fungal genera. All isolates were independently assessed three times using five experimental units per replicate. The *M. persicae* assays were conducted as an incomplete completely randomized design and analyzed within fungal genus using PROC GLM in SAS followed by a Bonferroni means separation procedure. The *A. tsugae* assays were conducted as a completely randomized block design and the lethal concentrations were determined using SAS PROC LOGIT with the logit switch in the model statement (SAS Institute 1996). For all fungal characterization studies, isolates were independently assessed three times for each temperature using four experimental units per replicate. The rates of growth, conidial production, and germination were analyzed within temperature as fixed effect ANOVA models using PROC GLM followed by a Student-Neuman-Keuls means separation in SAS (SAS Institute 1996).

## Results

### Entomopathogenic isolates recovered

Sixty-two isolates of entomopathogenic fungi were recovered from cadavers in the eastern USA, and 18 were recovered from southern China (Table 1). The fungal species recovered from the USA and China were similar, and the most prevalent fungi collected were *Lecanicillium lecanii* and *Isaria farinosa. *Both have known entomopathogenic associations with homopteran species (Milner and Lutton 1986; Kish et al. 1994). These fungi are commonly dispersed by wind and rain-splash, which may explain why they were most frequently observed. The remaining isolates were identified as *Beauveria bassiana* or *Fusarium* spp. *B. bassiana* is a cosmopolitan fungus with a broad host range and is commonly found in the soil (Goettel and Inglis 1997). *Fusarium* is also a soil fungus, and many species are phytopathogenic, occasionally occurring as weak entomopathogens (Humber 1997). In some cases, the observed fungi were not culturable. For example, in the spring 2007 sampling, 8% of *A. tsugae* were associated with *Beauveria*, but only nine were culturable. The fungal outgrowth on these cadavers was often yellow, which is a sign of colony aging based on the production of the secondary metabolite tenellin (Khachatourians and Qazi 2008), and senescence may be an explanation for the inability to culture those.

### Entomogenous isolates recovered

The list of fungal genera recovered from *A. tsugae* cadavers are presented in Table 2. The most common were *Alternaria* and *Cladosporium.* These fungi are commonly associated with aphid-infested plants (Agrios 1998), though some studies have identified entomopathogenic strains within both of these genera (Hatzipapas 2002; Abdel-Baky and Abdel-Salam 2003). In addition, these fungi quickly colonize insect cadavers, including those dead from senescence and those killed by fungal pathogens (Hatting et al. 1999), thus their presence may be obscuring the effect of other pathogens. In general, the proportion of fungi recovered from southern China were similar to those from the eastern USA, with the exception of *Acremonium* spp., which were associated with 13.8% of *A. tsugae* cadavers from China. Some species of *Acremonium* have been identified as entomopathogenic, including *Acremonium larvarum* and an *Acremonium* sp. ([Bibr bibr40]; Steenberg and Humber 1999). While it was not possible to isolate and perform bioassays of all putative pathogens from the fungal group, future studies investigating their impact on *A. tsugae* and their interaction with the entomopathogenic fungi isolated could explain their high abundance on *A. tsugae* cadavers.

### *Myzus persicae* bioassays

**Figure 1.**
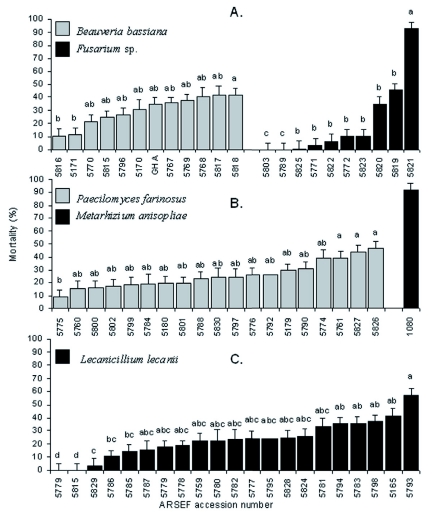
Virulence of entomopathogenic fungi against *Myzus persicae.* Values with the same letter within fungus are not significantly different using a Bonferroni means separation procedure at the α = 0.05 level of significance. ARSEF is the USDA Agricultural Research Service collection of Entomopathogenic Fungi. GHA is the active ingredient of BotaniGard, Laverlam International Corporation, Butte, MT. High quality figures are available online.

When the entomopathogenic isolates were screened against *M. persicae*, mortality ranged from 0–86% (Figure 1). When grouped by fungal species, mortalities were normally distributed for *B. bassiana*, *L. lecanii* and *I. farinosa* (p = 0.16, 0.25, and 0.11, respectively). These results reflect a broad range of efficacy within species. Mortalities among the *Fusarium* sp. isolates were not normally distributed (p = 0.02). Overall, 70% of these resulted in < 10% aphid mortality, while one isolate, 5821, demonstrated the highest efficacy (86%) (Figure 1A). This indicated that there was great diversity in the entomopathogenic capacity within the *Fusarium* isolates recovered. Similar rates of mortality were observed for all *B. bassiana* isolates, ranging from 10–35% (Figure 1A). Isolate 5817, collected in central Massachusetts, and 5818, from southern Connecticut, had the highest mortality, each resulting in 40% mortality, one-third higher than the commercial strain, GHA, which was equal in mortality to the others.

Among the 19 *I. farinosa*, mortality ranged from 10–43% (Figure 1B). Isolates were statistically similar (p > 0.05) with respect to mortality. The virulence of the *I. farinosa* isolates, with the exception of 5775, were statistically identical, thus 5826 and 5827 were selected for further study because they were from different geographic origins. Mortality rates of 6–54% were obtained among the *L. lecanii* isolates (Figure 1C). Statistically significant differences were found among the isolates (p < 0.0001), two of which produced > 50% mortality.

Based on the *M. persicae* bioassays, 10 isolates obtained from *A. tsugae* and two reference strains were selected for further efficacy studies against *A. tsugae* including: four *L. lecanii*, three *B. bassiana*, two *I. farinosa*, one *Fusarium* sp., one *M. anisopliae* 1080, and GHA.

**Table 2.**
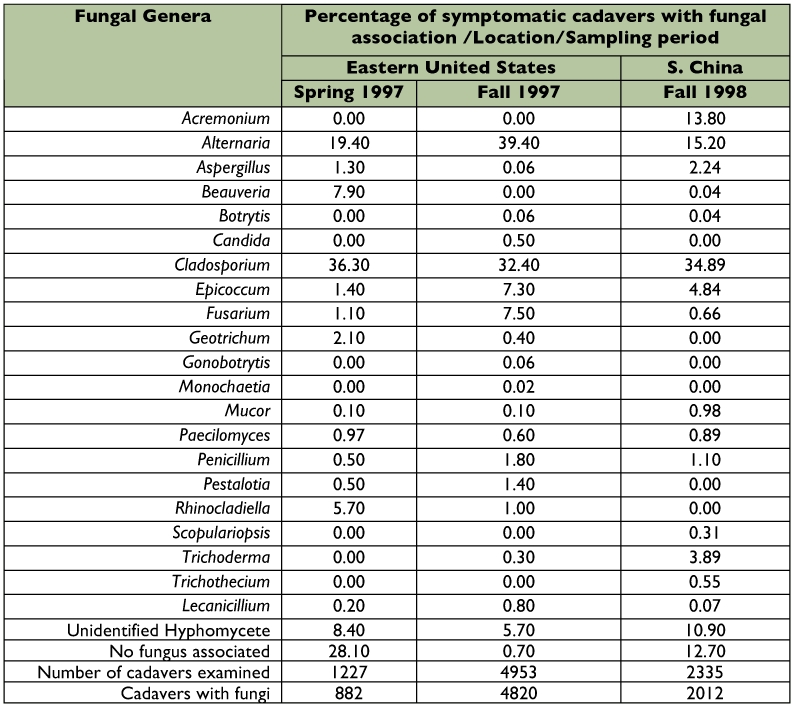
Percentage relative abundance of fungal genera associated with dead *Adelges tsugae* in the eastern US and southern China.

### *Adelges tsugae* bioassays

The 12 isolates tested were pathogenic to *A. tsugae* (Table 3), with mortality varying from 32–84% at the highest concentration used. The highest mortality was observed with 1080, which killed 84% of *A. tsugae* at an application rate of 1 × 10^8^ conidia per ml. The GHA strain and both *I. farinosa* caused the lowest mortality, while the mortality rates of the remaining isolates could be separated into three groups. The highest mortality rate was obtained from 5798, an *L. lecanii* from Massachusetts, followed by 5170 and 5824, a *B. bassiana* from Massachusetts and an *L. lecanii* from Virginia, respectively. There were no significant differences in the LC_50_ values for the remaining five (p > 0.05). Fungal outgrowth of the test fungi were obtained and confirmed to fungal species from > 80%) of all cadavers examined 11 d post application. The *A. tsugae* assays broadly separated the fungal isolates tested with respect to efficacy. The bioassay system used, however, was a complex system using field-collected branchlets. The types and abundance of microorganisms associated with the test branchlets could not be standardized. Therefore, *A. tsugae* mortality was quantified on new plant growth only. This ensured that mortality assessment was conducted on the summer aestivating generation only, and not on individuals from previous generations that had died, but remained on the tree. The most virulent against *A. tsugae* was 5170, which had a log LC_50_ of 7.09 (Table 3). Statistically, 5170 was more virulent than the three other *B. bassiana* tested and the third most virulent overall. 5818 and 5796 were not statistically different (p > 0.05), and the least virulent was GHA, which was significantly less virulent (p < 0.001) than the others. The most virulent *L. lecanii* was 5798, which was the second most virulent overall, followed by 5165, which was the fourth most virulent overall and was statistically non-resolvable from the other *L. lecanii.*

Neither *I. farinosa* isolate demonstrated strong virulence. The typical field application rate was comparable to the calculated LC25 for both. Extrapolation of the logit model estimated their LC_50_ values to be roughly 10 times higher than a typical field application rate of 1 × 10^7^ conidia per ml. Despite these results, *I. farinosa* was the second most frequently recovered fungus from the *A. tsugae* cadavers in the eastern USA collections and the most frequently recovered fungus from China.

The *Fusarium* sp. tested, 5821, was not as virulent against *A. tsugae* as it was against *M. persicae*, and it was not statistically different from the two least virulent *L. lecanii* or the least virulent *B. bassiana* recovered from *A. tsugae.* The LC_50_ value for 1080 was found to be the lowest for *A. tsugae* (7.9 × 10^5^ conidia per ml); 200 times lower than the typical field application rate of the industrial product BotaniGard. This was also the most efficacious against *M. persicae.*

**Table 3.**
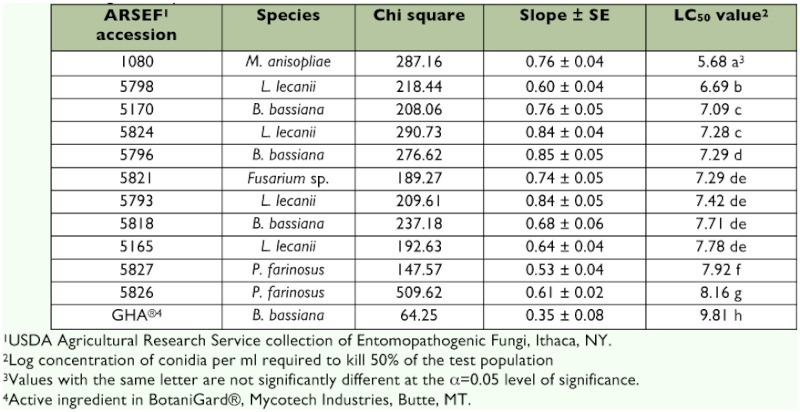
Results of bioassay testing of selected fungal isolates against *Adelges tsugae* applied to the Logit model. LC_50_ values are in log conidia per ml.

Based on the *A. tsugae* efficacy trials, four isolates were superior: *M. anisopliae* 1080, *B. bassiana* 5170 and 5796, and *L. lecanii* 5793.

### Characterization of fungi for growth and sporulation

**Figure 2.**
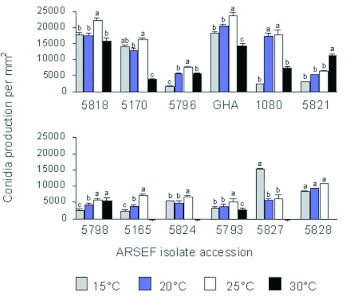
Rate of the production of conidia for all isolates tested against *Adelges tsugae.* ARSEF is the USDA Agricultural Research Service collection of Entomopathogenic Fungi, Ithaca, NY. GHA is the active ingredient of BotaniGard, Laverlam International Corporation, Butte, MT. High quality figures are available online.

In general, isolates had the fastest growth rates at 20 and 25° C (Table 4). The rate of growth for all isolates at 25° C was consistent for the first 10 d, but decreased significantly (p < 0.0001) by day 15. This is likely the result of growth restriction of the fungus caused by the limited size of the Petri dish. For this reason the rate of growth calculation only includes data up to day 10. Isolates of *B. bassiana* and *M. anisopliae* survived and grew at 30° C: however, *I. farinosa* 5826 and 5827 and *L. lecanii* 5165, 5793, and 5824 were unable to form colonies at this temperature and did show further growth when plates were removed at 20 d and placed at 22° C. When growth rates were compared at 25° C, the industrial strain GHA and *I. farinosa* 5827 grew significantly slower (p < 0.0001) than the other isolates. The rate of growth, however, does not take into account the productivity of the isolate. For this reason, the production of conidia was also measured. *B. bassiana* GHA, 5818, and 5170, produced the most conidia at all temperatures (Figure 2). In most cases, the production of conidia was highest at 25° C. Exceptions to this were *I. farinosa* 5827, which was most productive at 15° C, and *Fusarium* sp. 5821, which was most productive at 30° C. Excluding these isolates, spore production consistently increased from 15 to 25° C and then decreased at 30° C.

**Figure 3.**
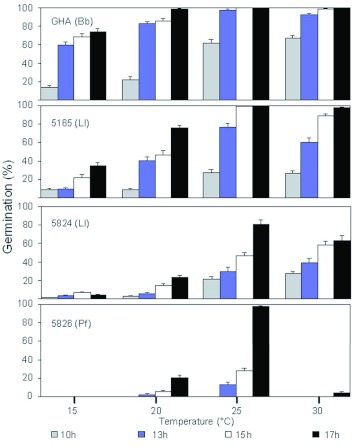
Germination rates for the fastest germinating isolate (GHA, *Beauveria bassiana*), the slowest germinating isolate (ARSEF 5826, *Isaria farinosa)* and two isolates for development as biological control agents (ARSEF 5265, *B. bassiana;* ARSEF 5824, *Lecanicillium lecanii*). GHA is the active ingredient of BotaniGard, Laverlam International Corporation, Butte, MT. Bb = *B. bassiana*, LI = *L. lecanii*, and If = *I. farinosa.* High quality figures are available online.

**Figure 4.**
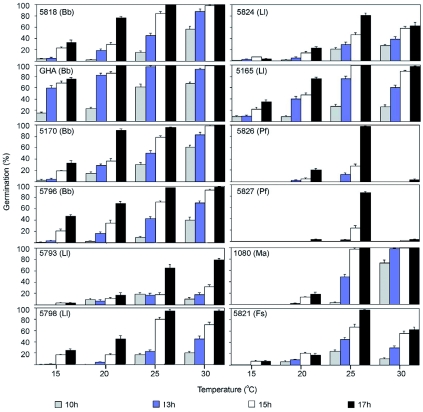
Germination rates for the fungal isolates tested in the characterization portion of this study. Numbers within boxes indicate the USDA Agricultural Research Service collection of Entomopathogenic Fungi accession numbers. GHA is the active ingredient of BotaniGard, Laverlam International Corporation, Butte, MT. Bb = *Beauveria bassiana*, LI = *Lecanicillium lecanii*, If = *Isaria farinosa*, Fsp = *Fusarium* sp., and Ma = *Metarhizium anisopliae.* High quality figures are available online.

In addition to growing and sporulating at field temperatures, these fungi must be able to germinate rapidly to colonize *A. tsugae.* The germination rates of 5165, 5824, 5826, and GHA are presented in Figure 3. The figure including germination rates of all test isolates at the four temperatures is shown in Figure 4. Overall, GHA spores germinated fastest at all temperatures tested, reaching nearly complete germination within 13 h at 25 and 30° C (Figure 3). Four isolates, *L. lecanii* 5165 and 5824 and *I. farinosa* 5826 and 5827 showed a general inability to tolerate 30° C. The *L. lecanii* were capable of germination at 30° C, but none of the four were able for form colonies at this temperature.

## Discussion

A total of 79 culturable entomopathogenic fungi were recovered from 8,515 *A. tsugae.* These fungi, *L. lecanii, I. farinosa, B. bassiana* and *Fusarium* spp., have a global distribution and are known pathogens of insects (Booth 1972; Humber 1997). Gouli et al. (1997) identified high levels of *Cladosporium* and *Alternaria* among populations of *A. tsugae.* These fungi are sooty molds associated with homopteran honeydew (Agrios 1988), and some species within these genera are known to be plant pathogens (Bustan et al. 2008), while other species have been documented as insect pathogens (Hatzipapas et al. 2002; Abdel-Baky and Abdel-Salam 2003). Their presence is relevant for the future development of a mycoinsecticide because the candidate isolate must be aggressive enough to overcome these rapidly-growing fungi. This may explain the efficacy of 1080. Although it was not initially isolated from *A. tsugae*, it is a soil fungus exhibiting rapid growth characteristics. Likely the most successful candidate strains will be those capable of overcoming the influence of antagonistic fungi.

**Table 4.**
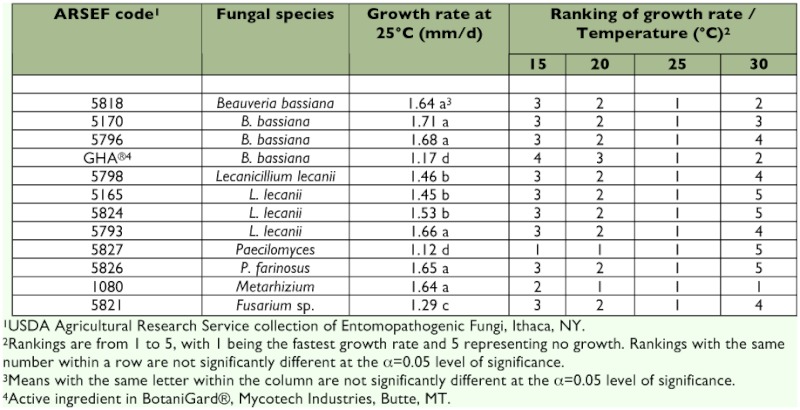
Rate of growth of fungal isolates, pathogenic to *Adelges tsugae*, grown for 20 d at different temperatures in the dark.

The efficacy of the selected entomopathogenic isolates against *A. tsugae* varied, as did their growth, germination and sporulation characteristics. GHA was found to have superior germination and sporulation characteristics, however, it was the least efficacious against *A. tsugae.*

The microclimates of the new and old growth of hemlock branchlets are unique with respect to each other. When trees no longer have new growth, *A. tsugae* populations rapidly decline (McClure 1991). For this reason, the *A. tsugae* bioassay testing was performed on new growth collected from healthy infested hemlock trees. While this allowed for a clear assessment of mortality and the resolution of relative efficacy among isolates, the assessment of the performance of these fungi on a large scale is still needed.

**Table 5.**
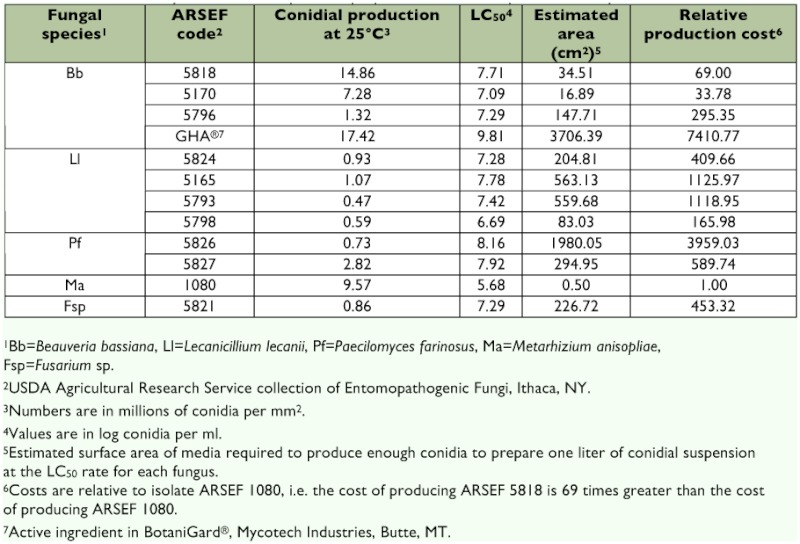
Estimated colony surface area required to prepare one liter of suspension at the predicted LC_50_ rate.

The base-line characteristics for a fungal-based biopesticide to manage *A. tsugae* require that the selected isolate have rapid germination to infect the host quickly, and cause mortality at field temperatures. It should also possess characteristics that make it suitable for mass-production, including a rapid growth rate and good sporulation. In general, for the purposes of commercial fungal spore production, complex substrates such as molasses, corn steep liquor, and various grains are used to obtain higher levels of sporulation than can be achieved on artificial medium (Jackson et al. 1997; Wraight et al. 2001). In this study, however, an artificial medium was selected to compare the relative production capacities of the fungal isolates. Based on this comparison, a relative cost of scale that is based on the amount of surface area of agar-based medium required to produce one liter of fungal material at the LC_50_ rate (Table 5) is proposed. Overall, *M. anisopliae* 1080, *B. bassiana* 5170 and 5818, and *L. lecanii* 5798 required the lowest amount of medium to produce enough material for one liter at the LC_50_ rate.

Although the commercial production of a mycoinsecticide would not be based on an agar-based production system, a comparison based on these data was conducted. When the surface area required to produce enough material for 1 l of the LC_50_ rate for these fungi was estimated, the capacity to produce virulent-conidial production relative to the most virulent, 1080, showed that four isolates would be the most suitable for further development as biological control agents. These were *M. anisopliae* 1080, *B. bassiana* 5170 and 5818, and *L. lecanii* 5798. The use of different formulations for these isolates may also improve their overall efficacy under field conditions (Daoust et al. 1983; Pan et al. 1988; Lomer et al. 1993; McClatchie et al. 1994; Alves et al. 1997; Evans 1999).

This study provides baseline information on the fungi associated with *A. tsugae* and their ability to cause mortality against low density populations of aestivating sistens. This generation was selected because no protocol for rearing *A. tsugae* has been developed and it is the only generation that can be reliably field-collected without contaminating individuals from previous generations. Both the progrediens and sistens remain throughout their life on hemlock at the site where they initially feed as crawlers, thus multiple generations of live and dead *A. tsugae* can occupy the same part of the tree. This makes the actual estimation of mortality difficult because individuals killed from a test treatment may be difficult to resolve from dead *A. tsugae* from previous generations. The spore production characterization studies showed that some isolates performed better at the cooler temperatures, indicating that these may be more active during cooler periods, when the other life forms of *A. tsugae* are present. Due to this, the use of isolates active at cooler temperatures for field applications in the spring or fall periods should be considered. Further, it should be considered that while our study demonstrates the ability of fungi to cause mortality in populations of *A. tsugae*, the field application of a mycoinsecticide may not yield the same results and any effective application of fungi for *A. tsugae* management would require a novel approach.

## Abbreviations

ARSEF: Agricultural Research Service Collection of Entomopathogenic Fungi;
SDAY: Sabouraud dextrose agar and yeast;
GHA: *Beauveria bassiana* Strain GHA, the active ingredient in BotaniGard
